# Similarities between maternal and fetal RR interval tachograms and their association with fetal development

**DOI:** 10.3389/fphys.2022.964755

**Published:** 2022-11-21

**Authors:** Namareq Widatalla, Ahsan Khandoker, Mohanad Alkhodari, Kunihiro Koide, Chihiro Yoshida, Yoshiyuki Kasahara, Yoshitaka Kimura, Masatoshi Saito

**Affiliations:** ^1^ Graduate School of Biomedical Engineering, Tohoku University, Sendai, Japan; ^2^ Healthcare Engineering Innovation Center, Department of Biomedical Engineering, Khalifa University, Abu Dhabi, United Arab Emirates; ^3^ Cardiovascular Clinical Research Facility, Radcliffe Department of Medicine, University of Oxford, Oxford, United Kingdom; ^4^ Tohoku University Graduate School of Medicine, Sendai, Japan

**Keywords:** maternal-fetal RRI similarity, heart rate vaiability, very low frequency, fetal developement, fetal programming

## Abstract

An association between maternal and fetal heart rate (HR) has been reported but, so far, little is known about its physiological implication and importance relative to fetal development. Associations between both HRs were investigated previously by performing beat-by-beat coupling analysis and correlation analysis between average maternal and fetal HRs. However, studies reporting on the presence of similarities between maternal and fetal HRs or RR intervals (RRIs) over the short term (e.g., 5-min) at different gestational ages (GAs) are scarce. Here, we demonstrate the presence of similarities in the variations exhibited by maternal and fetal RRl tachograms (RRITs). To quantify the same similarities, a cross-correlation (CC) analysis between resampled maternal and fetal RRITs was conducted; RRITs were obtained from non-invasive electrocardiogram (ECG). The degree of similarity between maternal and fetal RRITs (bmfRRITs) was quantified by calculating four CC coefficients. CC analysis was performed for a total of 330 segments (two 5-min segments from 158 subjects and one 5-min from 14 subjects). To investigate the association of the similarity bmfRRITs with fetal development, the linear correlation between the calculated CC coefficients and GA was calculated. The results from the latter analysis showed that similarities bmfRRITs are common occurrences, they can be negative or positive, and they increase with GA suggesting the presence of a regulation that is associated with proper fetal development. To get an insight into the physiological mechanisms involved in the similarity bmfRRITs, the association of the same similarity with maternal and fetal HR variability (HRV) was investigated by comparing the means of two groups in which one of them had higher CC values compared to the other. The two groups were created by using the data from the 158 subjects where fetal RRI (fRRI) calculation from two 5-min ECG segments was feasible. The results of the comparison showed that the maternal very low frequency (VLF) HRV parameter is potentially associated with the similarity bmfRRITs implying that maternal hormones could be linked to the regulations involved in the similarity bmfRRITs. Our findings in this study reinforce the role of the maternal intrauterine environment on fetal development.

## Introduction

The fetal programming theory postulates that the maternal intrauterine environment can influence the offspring after birth and into adulthood. The theory implies that the upbringing of healthy adults is heavily dependent on maternal health and behavior during the prenatal period (Barker, 1995; Godfrey and Barker, 2001; Kwon and Kim, 2017). Hence, recognition of maternal factors that may affect fetal development during pregnancy can potentially enhance the quality of life of the fetus during adulthood.

Currently, fetal well-being and development are widely assessed by measuring fetal heart rate (HR) and HR variability (HRV) (Gonçalves et al., 2006a; Gonçalves et al., 2013; DiPietro et al., 2015). Fetal HR and HRV change throughout gestation (DiPietro et al., 2015) and they were found to be affected by fetal presentation (Gonçalves et al., 2014), fetal gender (Tendais et al., 2015), fetal health (Gonçalves et al., 2006b; Gonçalves et al., 2018), and fetal behavioral states (Nijhuis et al., 1982; Gonçalves et al., 2007). Fetal HR and HRV were also found to be influenced by different maternal-related factors such as respiration (Leeuwen et al., 2009), weight (Husin et al., 2020), exercise (Leeuwen et al., 2014; May et al., 2016), and sleep (Dipietro et al., 2021), however, the mechanisms underlying such influences are not fully understood yet. The placenta, which is the point of connection between the mother and fetus, is expected to play a major role in these influences. In addition, according to previous studies that addressed maternal-fetal HR coupling or interaction (Leeuwen et al., 2003; Leeuwen et al., 2009; Gonçalves et al., 2016; Khandoker et al., 2019), it is highly implied that the mechanisms leading to changes in fetal HRs (fHRs) can be understood through maternal HRs (mHRs).

It has been reported that fHRs have a diurnal rhythm because they decreased and increased at night and daytime, respectively. The diurnal changes that were observed in fHRs were in harmony with mHRs (Vries et al., 1987; Lunshof et al., 1998). The simultaneous increase and decrease in maternal and fetal HRs suggest an interaction between both, but the correlation between the same remains elusive over a short period. Until now, there is a discrepancy in the literature regarding the association of fHR with mHR over the short term, for example, J. Dipietro et al. (Dipietro et al., 2004; Dipietro et al., 2006) investigated the temporal associations between maternal and fetal HRs over a 50-min period but reported that there were no associations between maternal and fetal HRs. On the other hand, in a recent study by J. Dipietro et al. (2021), a little temporal coupling between maternal and fetal HRs was reported, but only at certain maternal sleep stages.

So far, the reason behind maternal-fetal HR coupling occurrence and its association with fetal development are unknown. In addition, there is inconsistency in the literature regarding the cause behind maternal-fetal HR coupling and the physiological pathways that may affect it. Previously, it was addressed that maternal-fetal HR coupling was affected by respiration (Leeuwen et al., 2009), on the contrary, another study by F. Marzbanrad et al. (2015) reported that respiration was not related to coupling. Concerning the causal effect, P. Leeuwen et al. (2009) mentioned that the cause behind coupling is still unknown and it could be mediated by the fetal auditory system. On the other hand, J. Dipietro et al. (2021) suggested that coupling may occur due to a physiological process that mediates both maternal and fetal HRs.

The previously mentioned studies adopted different mathematical methods to quantify maternal-fetal HR coupling or correlation, but none of them tried to look at maternal and fetal RR interval tachograms (RRITs) after normalizing and plotting them together in one figure to investigate the possibility of finding similarities between both. Due to the high dynamicity of the heart, normalizing RRITs calculated from long recordings (e.g., more than 15-min) may make finding such similarities difficult, especially if high fluctuations in RRITs were present within the record. Therefore, choosing a proper window size to investigate the presence of similarities between maternal and fetal RRITs (bmfRRITs) is critical.

Due to the limited knowledge in the field, identification of mechanisms that mediate maternal and fetal HRs is challenging. Nevertheless, understanding the patterns in which they interact can potentially assist in identifying the cause. In addition, understanding the same patterns may potentially contribute to the clinical diagnosis of fetal distress and pregnancy complications.

In this study, for the first time, we report the presence of similarities bmfRRITs. RRITs were obtained from simultaneous non-invasive measurements of maternal and fetal electrocardiogram (ECG). The presence of similarities bmfRRITs. RRITs was confirmed by normalizing maternal and fetal RRITs and plotting them together in one figure. Occasionally, the same similarities were obvious by looking at oscillations occurring slowly in RRITs. After confirming the presence of similarities bmfRRITs., we opted for obtaining mathematical measures for them by using cross-correlation (CC) analysis. We hypothesized that the similarities bmfRRITs could be linked to fetal development, therefore, we performed a linear correlation analysis between our developed CC coefficients and GA. To get more insight into the physiological pathways or factors involved in the similarity bmfRRITs, we investigated the association of maternal and fetal HRV with GA as well. Also, we used CC coefficients to divide data into two groups to explore how maternal and fetal HR and HRV may change relative to the similarities bmfRRITs.

## Materials and methods

### Data collection

The study described in this manuscript was approved by the Tohoku University Institutional Review Board (Approval number: 2021-1-133). A total of 406 outpatient or in-patient pregnant women (GA: 19–40 weeks), who visited Tohoku University Hospital, Japan, for antenatal checkups or treatment of pregnancy-related illnesses, were recruited during 2009–2019 for different projects that were carried out at Tohoku University. The women were recruited after getting their informed consent. The 406 sample size reflects the number of participants who were recruited from among all pregnant women who visited the Tohoku University Hospital. Pregnant women who were recruited were at least 20 years old and could read and understand the written informed consent in Japanese.

Before recruiting the participants, an obstetrician confirmed the schedule and location of the ECG measurements. Then a subject who met the selection criteria, mentioned above, was approached for recruitment after informing her about the research details and ECG measurements. Demographic data of the participants were collected such as age, height, and weight. Information regarding maternal health and medication along with fetal weight and health were recorded. Participants were asked to remain in a supine position and 12 electrodes were attached to their abdominal surface to obtain simultaneous records of non-invasive maternal and fetal ECG. The recordings lasted for 20-min, and the sampling frequency was 1 kHz. In this study, we analyzed data retrospectively.

### Data selection and fetal ECG extraction

The exclusion criteria for this study entailed: 1) fetuses who had records of medical complications when ECG data were collected, and 2) subjects with missing information regarding GA or fetal or maternal health. The total number of data that met the exclusion criteria was 211, hence, around 195 data were considered for analysis in this study.

Extraction of fECG from maternal ECG (mECG) was conducted by using a MATLAB 2008b code. The code extracts fECG based on blind source separation with reference (BSSR) which is described in detail in (Sato et al., 2007). fECG extraction was performed for at least 5-min per subject. No particular procedures were followed to assess the quality of the raw abdominal signals to select a 5-min segment for fECG extraction. A signal’s quality was considered good if the software was able to extract clear fECG signals. Clear fECG signals made fetal R peak detection easy. A window size of at least 5-min was chosen mainly to accommodate for the very low frequency (VLF) band (0.0033–0.04) Hz. Due to technical limitations related to the quality of the raw abdominal signals, it was difficult to perform analysis on more than 5-min lengths.

fECG extraction attempts were carried out in chronological order. Hence, fECG extractions were done starting from the beginning of the ECG records, if the software failed to extract fECG from the selected segments, extractions of the next 5-min segments were attempted. If the extraction was not successful in any 5-min segment, the data were excluded. As a result of the latter steps, fECG signals were extracted at the beginning, middle, or end of the recording.

In the initial extraction attempts, the total number of 5-min segments (1 segment per 1 subject) that were extracted from the 195 subjects was 172 (age: 22–45 years old (34 ± 5.3), GA: 19–40 weeks (30 ± 6.1). Extraction of fECG from the rest of the 23 subjects was not possible due to noise (e.g maternal myoelectric and environmental noise). After analysis of the 172 segments, an additional 5-min segments were extracted to get more insights into the similarity bmfRRITs and its correlation with HRV. Extraction of additional segments of 5-min was possible in 158 cases (age: 22–44 years old (34 ± 5.3), GA: 19–40 weeks (30 ± 6.2)). With the second fECG extraction, the total number of 5-min segments extracted per subject was two in 158 subjects. The additionally extracted 5-min segments did not overlap with the previously extracted segments. Extraction of two segments of 5-min from all 172 subjects was not possible due to noise in the data, also, around three data sets had recordings of less than 10-min.

Around 44 pregnant women had no records of medical or obstetric complications, however, the rest of the subjects had at least one complication, more details are found in (Supplementary Table S1). Also, the distribution of GA of the 172 subjects is provided in (Supplementary Figure S1).

## Similarity quantification with cross-correlation analysis

### RRI and HRV calculation

To investigate the presence of similarity bmfRRITs and further investigate the similarity’s association with fetal development, we calculated RRI and HRV. To obtain RRITs from ECG, R peaks were detected by using the “findpeaks” function in MATLAB 2020b and the code is described in (Mathworks, 2022), detected R peaks were verified visually to ensure that the code detected R peaks only, example of detected R peaks is demonstrated in (Supplementary Figure S2). R peaks were detected in maternal and fetal ECG signals which were captured at 1 kHz. The “findpeaks” function detects peaks based on a threshold value, here the threshold value was adjusted based on the R peak amplitudes that varied among subjects. The “findpeaks” provides the location of the detected peaks along with their amplitudes. Following R peak detections, the time difference between two successive R peaks was calculated to obtain RRI signals or RRITs. By using RRI, maternal and fetal HRV parameters were calculated.

Time and frequency-based HRV analysis was performed in MATLAB. For HRV analysis, original non-resampled RRI data was used and abnormal sinus RRI values were corrected manually by replacing them with preceding or subsequent RRI values. Time-based HRV analysis entailed calculations of the average RRI, standard deviation (SD) of normal RRI (SDNN), and SD of HR (SDHR). Frequency-based analysis was done by using the Lomb-Scargle periodogram with considering the following bands:

mECG: VLF: (0.0033–0.04) Hz, LF: (0.04–0.15) Hz, high frequency (HF): (0.15–0.4) Hz.

fECG: VLF (0.0033–0.03) Hz, LF: (0.03–0.2) Hz, HF (0.2 - 2) Hz.

mECG bands were chosen based on previously defined bands for human adults (Task Force of the European Society of Cardiology the North American Society of Pacing Electrophysiology, 1996). Since up until now there are no well-defined bands for fECG, we used bands that were used for infants (Chatow et al., 1995; Thu et al., 2019).

### Similarity trend analysis

To check for the presence of similarity epochs bmfRRITs, the values of RRI signals were normalized by using Eq. 1:

$$Normalized\;RRI=\frac{RRI-mean\;RRI}{\max\left(RRI-mean\;RRI\right)}$$ (1)

After normalization, maternal and fetal RRITs were plotted together in one panel to visualize the similarities bmfRRITs.

### Cross-correlation coefficient calculation

**FIGURE 1:**
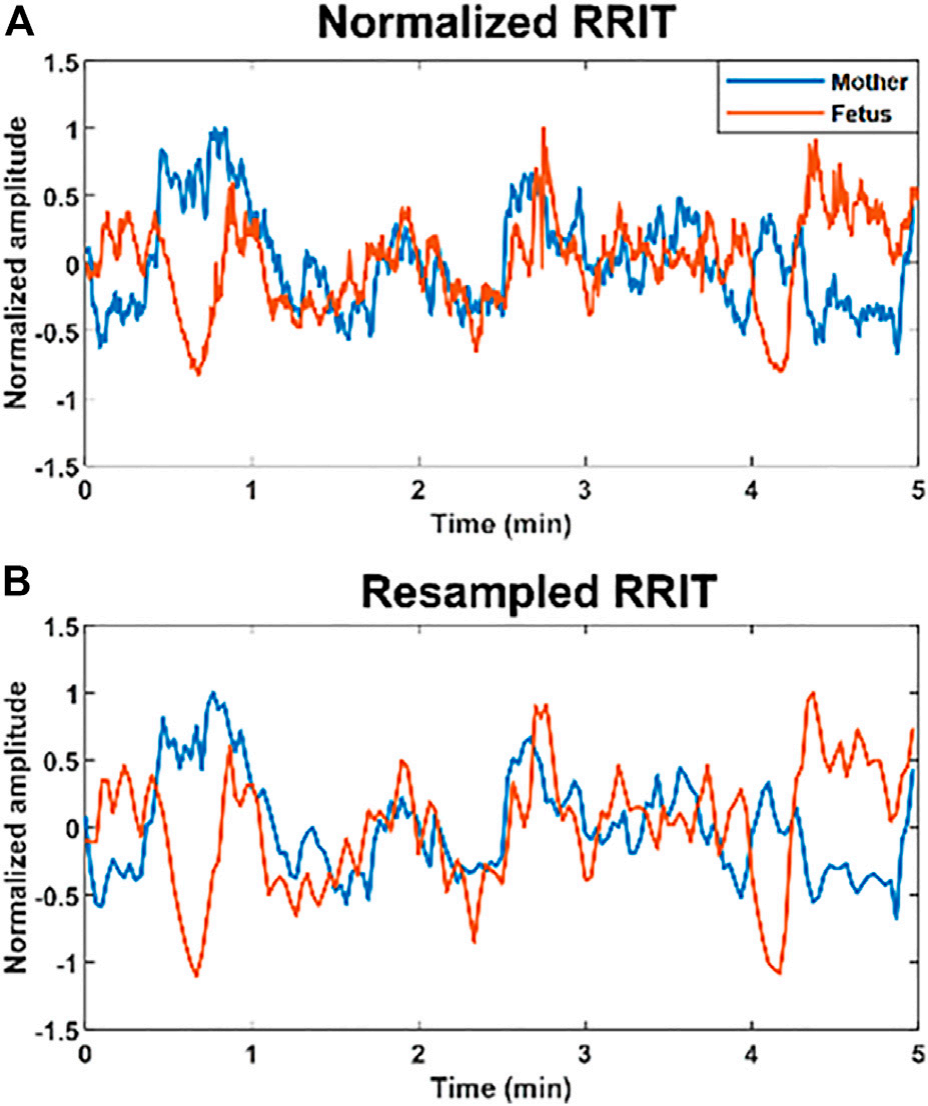
Example of normalized and resampled maternal and fetal RR interval tachograms (RRITs). **(A)** The figure shows an example of maternal (blue) and fetal (orange) RRITs before resampling, the amplitudes are normalized in this figure by using Eq. 1. **(B)** The figure shows resampled RRITs, resampling was done by taking the average of RRI per 2 s.

To obtain a similarity score or a mathematical measure of the similarity bmfRRITs, we performed CC analysis in MATLAB 2020b. CC analysis measures the similarity between two signals at different time lags, more details can be found in (xcorr, 2022). Due to the difference in the range of maternal and fetal RRI values, the number of maternal RRI samples per 5-min segment was lower than that of the fetus. Hence, to unify the lengths of maternal and fetal RRI signals per 5-min segment, we resampled both at 0.5 Hz. Resampled RRI signals were calculated by taking the average of RRI per 2 s. Resampling per 2 s (2000 samples) yields a signal with 150 samples for a 5-min signal (300,000 samples), $\frac{300,000}{2000}=150$ . Example of a resampled signal is demonstrated in (Figure 1); (Supplementary Figure S2) provides more information about resampling. Resampled maternal and fetal RRI signals were then normalized per subject by using Eq. 1.

After resampling and normalization, the resampled RRI signals (with 150 samples) were divided into 15 segments to calculate CC. CC coefficients were calculated per 10 samples by using the “xcorr” function in MATLAB; CC values were calculated with a zero-time lag. We opted for calculating CC per 10 samples rather than the whole 150 samples to capture transient changes in the similarity. CC analysis of the whole resampled RRI signal (150 samples) may lead to an underestimation of the similarity. Next, the overall similarity per case (5-min segment) was estimated by taking the average of the 15 coefficients. Here, we adopted four different methods to estimate the overall similarity bmfRRITs. We used different methods for CC coefficient calculations because, so far, it is unknown what could be a good way to quantify similarity bmfRRITs to get insights into fetal development. We calculated our four CC coefficients as follows:

CC1: this coefficient was calculated by taking the absolute average of the 15 coefficients (CC1 is not normalized). CC1 provides a rough score for the similarity, the higher the CC1 value is, the higher the degree of similarity bmfRRITs.

CC2: this coefficient was calculated by taking the non-absolute average of the 15 coefficients (CC2 is not normalized). CC2 quantifies the similarity bmfRRITs by considering directionality (whether maternal and fetal RRIs are changing in the same or opposite directions). Since CC2 is a non-absolute mathematical average of 15 CC coefficients, the sign of the CC2 value reflects the dominant similarity trend, positive or negative, within a 5-min segment.

CC3: to calculate this coefficient, the “normalized” option of the “xcorr” function was applied in MATLAB when the 15 coefficients were calculated. After that, the absolute average of the 15 coefficients was calculated. The meaning of CC3 is similar to that of CC1.

CC4: the 15 coefficients were calculated similarly to CC3 (with the “normalized” option in MATLAB), then the non-absolute average of the 15 coefficients was calculated. The meaning of CC4 is similar to that of CC2.

A summary of CC1, CC2, CC3, and CC4 calculations is provided in (Supplementary Figure S3). We performed a brief comparison among our derived coefficients based on their potential linkage to fetal development. The linkage was assessed by performing a linear correlation analysis between the CC coefficients and GA.

### Data classifications based on CC1 and CC3

**FIGURE 2:**
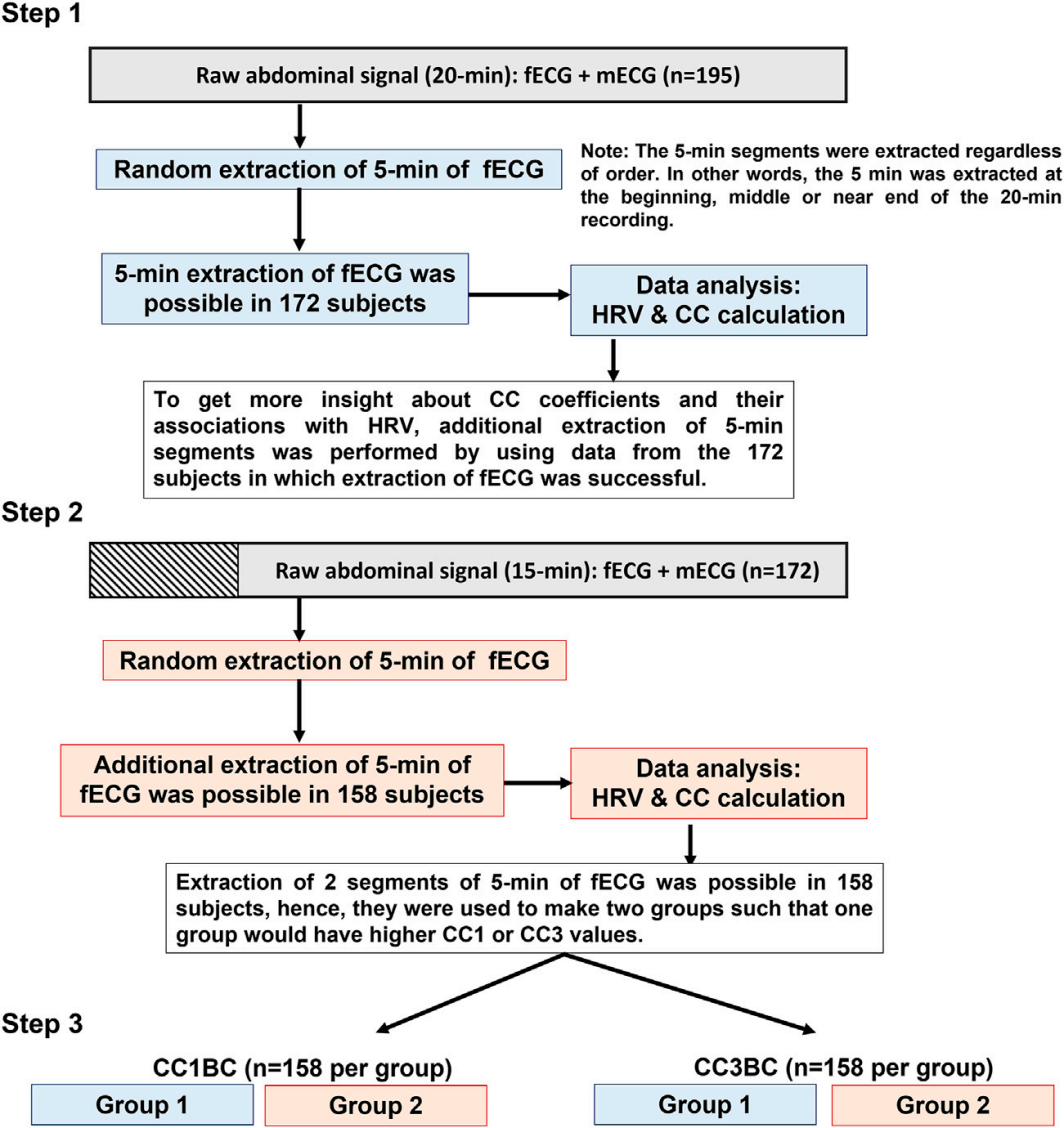
Summary of data analysis. The flowchart provides a graphical summary of the steps that were followed to analyze the data. In Step 1, 5-min extraction of fetal electrocardiogram (fECG) was successful in 172 out of 195 subjects. In step 2, additional extraction of 5-min segments was successful in 158 out of the 172 subjects. Both in step 1 and step 2 cross-correlation (CC) and maternal and fetal heart rate variability (HRV) analyses were performed for the extracted 5-min segments. In step 3, a comparison of means analysis was performed to compare between group 1 and group 2 in terms of maternal and fetal HRV analysis. Group 2 has higher CC1 or CC3 values compared to group 1.

To get more physiological insight into the similarity bmfRRITs, we made two groups by using the 2 extracted segments of 5-min (from the 158 subjects) to compare their HRV features. The comparison analysis was carried out twice. In the first comparison, the data were classified based on the CC1 coefficient (CC1-based classification (CC1BC)) and in the second comparison, data were classified based on the CC3 coefficient (CC3-based classification (CC3BC)). Group 2 had higher values of CC1 or CC3 compared to group 1. The main purpose of this analysis is to see if there will be significant differences in HRV between the two groups due to the effect of CC1 or CC3 (Figure 2). shows a summary of data extraction and CC classification.

Here, we based our classification on CC1 and CC3 only because, mathematically, they provide a stronger measure for the overall similarity bmfRRITs compared to CC2 and CC4. In CC2 and CC4, similarity quantification by using CC analysis might be underestimated due to the mathematical summation of negative and positive numbers. Also, data classification based on CC2 and CC4 was not done due to the complexity involved in the classification and interpretation of the results (more details are found in the discussion).

### Statistical analysis

Normality tests were conducted in MALTAB 2020b by using the One sample Kolmogorov-Smirnov test (kstest) and the Shapiro-Wilk test (swtest) (BenSaïda, 2014). Kstest revealed that all variables were non-normally distributed regardless of group. On the other hand, swtest revealed that some variables were normally distributed in both groups (group 1 and group 2), and others were normally distributed in one group only. Hence, we based our normality tests on the kstest only.

Correlation analysis between two variables was performed by using the spearman test.

Comparison of means was performed by using the Friedman test.

## Results

### Demonstration of similarities between maternal and fetal RRITs

**FIGURE 3:**
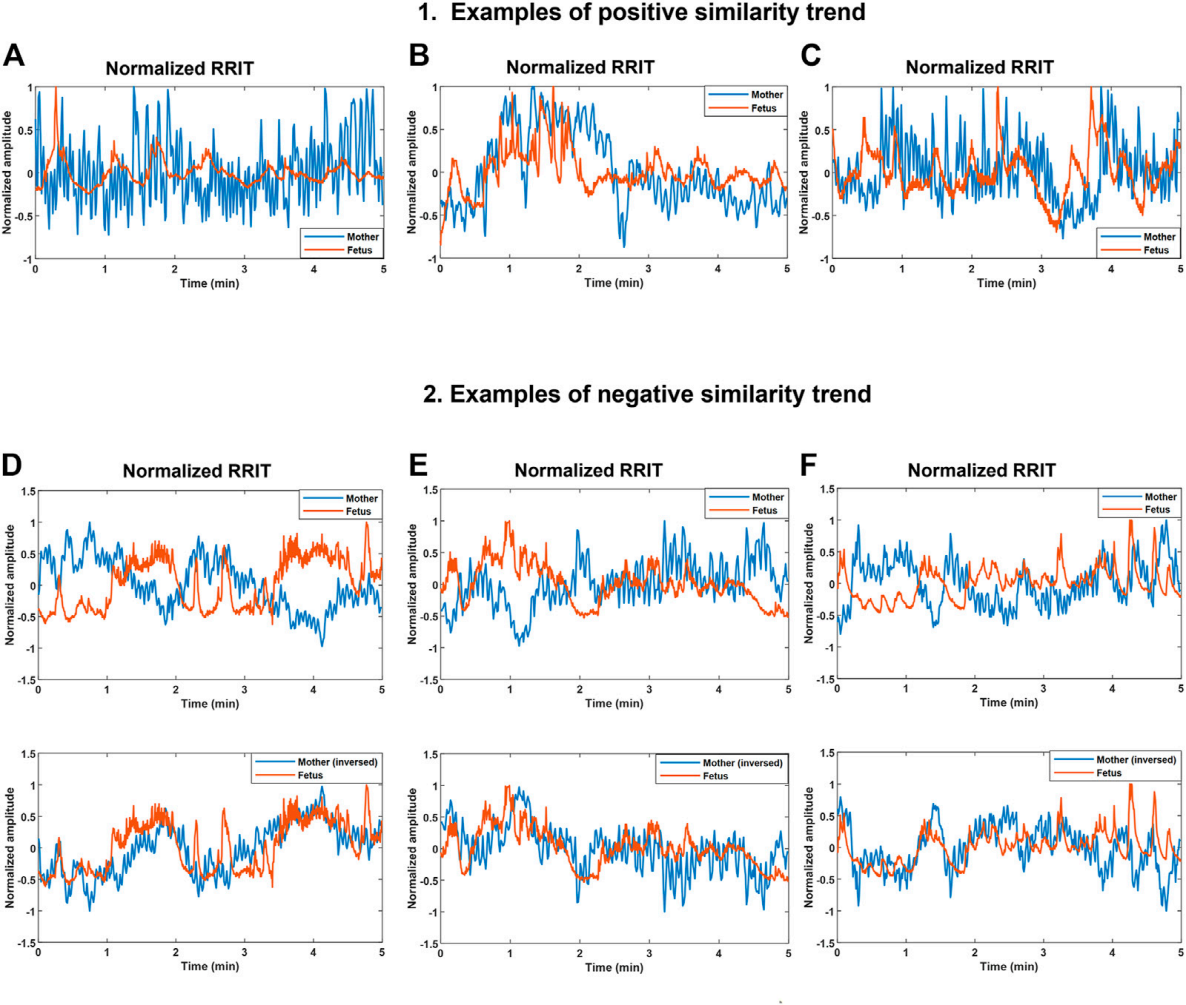
Demonstration of positive and negative similarity trends between maternal and fetal RR interval tachograms (bmfRRITs). Figures **(A–C)** show examples of positive similarity trends in which maternal (blue) and fetal (orange) RRITs change in the same direction. Figures D, E and F **(D–F)** show examples of negative similarity trends in which maternal and fetal RRITs change in opposing directions. The upper panels in Figures **(D–F)** show the original signals while the lower panels show the original fetal signal with the maternal signal inversed. **(A)** The record belongs to a mother who had no records of medical complications, gestational age (GA): 20 weeks. **(B)** The record belongs to a mother with a record of uterine/appendix disease, GA: 23 weeks. **(C)** The record belongs to a mother with a medical record of respiratory disease and uterine/appendix disease, GA: 20 weeks. **(D)** The record belongs to a mother with a medical record of autoimmune disease, gestational age (GA): 39 weeks. **(E)** The record belongs to a mother who had a blood disease, GA: 33 weeks. **(F)** The record belongs to a mother with no records of medical complications, GA: 2 weeks.

We hypothesized that changes occurring in maternal and fetal RRITs may share resemblances occasionally, hence, we aimed at investigating the possibility of finding similarities bmfRRITs. To achieve this, we collected RRITs signals from maternal and fetal ECG. Then, maternal and fetal RRITs were resampled, normalized, and plotted together (Figure 3).

Maternal and fetal RRITs were found to exhibit positive and negative similarity trends. In a positive similarity trend (Figures 3A-C), maternal and fetal RRITs change in the same direction. In (Figure 3A), it is noticeable that the LF and VLF oscillations exhibited by fetal RRI (fRRI) are similar to that of the maternal RRI (mRRI). In (Figure 3B), fetal and maternal RRI increased in synchrony before the first minute, and then, they decreased but with a time lag at around the second minute. Similarities bmfRRITs were found to exhibit time lags as demonstrated in (Figure 3C **)** over the period 2.7–4 min.

In negative similarity trends, maternal and fetal RRIs change in opposing directions (Figures 3D–F). The upper panels in (Figures 3D–F) show the original normalized RRITs and the lower panels show the same but with the maternal signal inversed. After inversing maternal RRITs, the similarities bmfRRITs are clearer. In (Figure 3D), the increase in fRRI at around the first minute was accompanied with a decrease in mRRI but with a time lag, the latter is made clearer in the lower panel after inversing the maternal signal. Another example of a negative similarity trend is demonstrated in (Figure 3E). In (Figure 3F), RRIs are changing in opposing directions over the period 0–3.5 min, however, the trend changes to positive afterward.

### Similarities are related to fetal development

We hypothesized that the similarities bmfRRITs that were demonstrated in (Figure 3) could be associated with fetal development. Hence, we opted for obtaining mathematical measures for the similarity bmfRRITs by using CC analysis in which we obtained four coefficients, CC1, CC2, CC3, and CC4. The linear correlation between the four coefficients and GA was calculated to investigate the association of similarity bmfRRITs with fetal development. The results of this analysis are shown in the upper rows of (Table 1) (the correlation coefficient with GA is represented as *r*).

**TABLE 1:** Summary of maternal and fetal HRV, CC coefficients and their correlations with GA, n = 172.

Feature	Correlation between CC coefficients and GA			
	Median (min – max)	(Mean ± SD)	*r*	
CC1	0.58 (0.11–2.5)	0.63 ± 0.32	0.40†	
CC2	−0.023 (−1.3–0.80)	−0.09 ± 0.36	−0.26†	
CC3	0.43 (0.21–0.71)	0.44 ± 0.10	0.19*	
CC4	−0.012 (-0.53–0.49)	−0.03 ± 0.18	−0.20†	
**Feature**	**Correlation between HRV and GA**			
	**Maternal Features**		**Fetal features**	
	**(mean ± SD) median (min-max)**	***r***	**(mean ± SD) median (min-max)**	***r***
RRI (ms)	760 ± 113	−0.03	412 ± 25	0.33†
	742 (530–1,125)		409 (354–512)	
SDNN (ms)	35 ± 14	0.35†	16 ± 6.9	0.56†
	31 (13–87)		15 (4.0–36)	
SDHR (bpm)	3.7 ± 1.4	0.39†	5.7 ± 2.5	0.49†
	3.5 (1.5–8.9)		5.2 (1.6–14)	
VLF (Ln)	6.2 ± 0.76	0.45†	4.5 ± 1.1	0.52†
	6.2 (4.1–8.2)		4.6 (1.8–6.8)	
LF (Ln)	5.1 ± 0.80	0.16*	4.3 ± 0.85	0.53†
	5.1 (3.3–7.7)		4.4 (1.4–6.1)	
HF (Ln)	4.7 ± 1.3	0.10	2.3 ± 0.81	0.53†
	4.7 (1.1–7.8)		2.4 (-0.46–4.2)	

*p < 0.05, †*p* < 0.005. Abbreviations: HRV, heart rate (HR) variability; GA, gestational age; CC, cross-correlation; RRI, RR interval; SD, standard deviation; SDNN, SD of normal RRI; SDHR, SD of HR; bpm, beats per minute; VLF, very low frequency power; LF: low frequency power; HF, high frequency power; *r*, spearman correlation coefficient.

**FIGURE 4:**
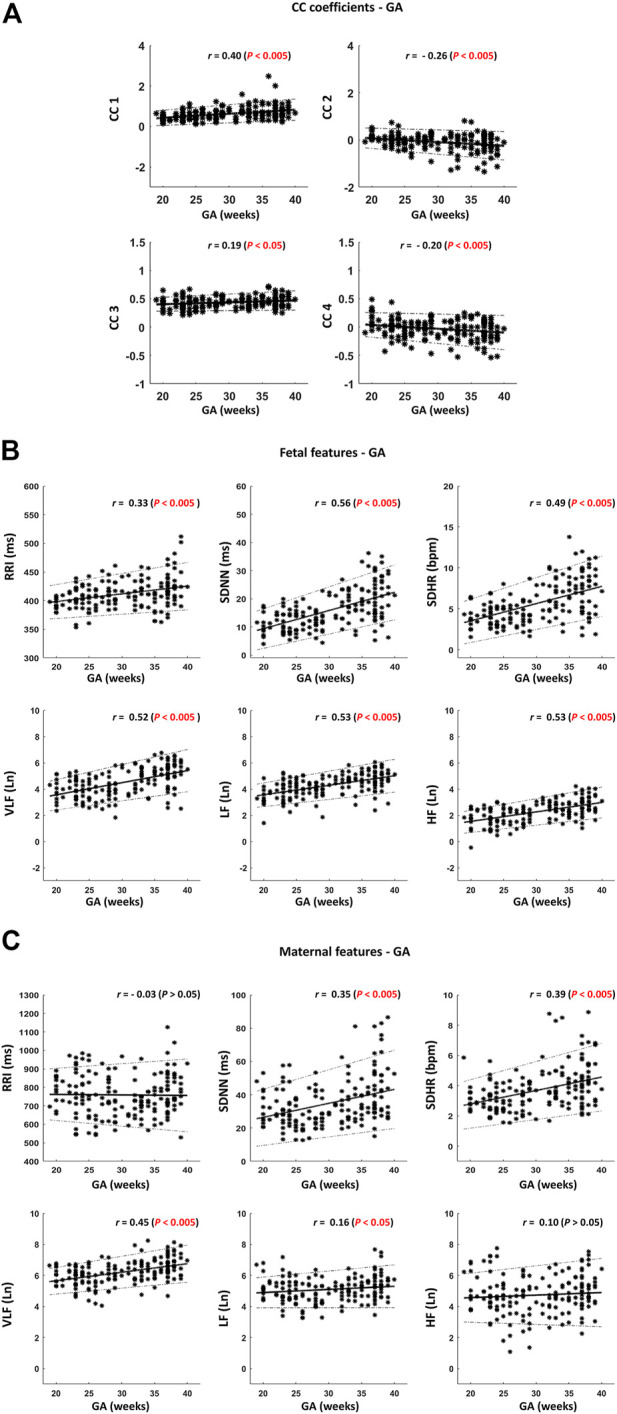
Scatter plots for visualizing the correlations described in Table 1. **(A)** Cross-correlation (CC) coefficients – gestational age (GA). **(B)** Fetal features—GA. **(C)** Maternal features – GA. Thick black lines indicate the best-fitting line. The dotted black lines indicate the 95% confidence interval (CI). The sample size is 172. *r* indicates Spearman correlation coefficients.

For further investigation, we calculated the linear correlations between fHRV and GA and between maternal (mHRV) and GA. The mean, SD, median, and ranges of HRV parameters along with their correlations with GA are shown in (Table 1). Graphical representations of the previously mentioned linear correlation analyses, CC coefficients—GA, fHRV—GA and mHRV—GA, are demonstrated in (Figure 4).

Among the four CC coefficients, CC1 was found to have the highest significant correlation with GA where r = 0.40 [(Table 1) and (Figure 4A)]. At advanced GA, it was revealed that the negative similarity trend prevails, and this is indicated in CC2 and CC4 with r values of −0.26 and −0.20, respectively (Table 1) and (Figure 4A). The results of fHRV—GA correlation analysis showed that there was a significant increase in the associations of fetal HRV and RRI with GA, (Table 1) and (Figure 4B). The mHRV - GA correlation analysis showed that mHRV, VLF, SDHR, and SDNN were significantly correlated with GA (Table 1) and (Figure 4B). Also, LF was found to be significantly correlated with GA but with a lower r value compared to VLF, SDHR, and SDNN (Table 1) and (Figure 4B).

### Data classifications based on CC1 and CC3

We aimed at investigating how HRV is associated with the similarity bmfRRITs (or CC coefficients) to identify mechanisms or physiological pathways that could be associated with the same similarity. Hence, we extracted additional 5-min segments to make two groups such that one group would have a higher similarity score or a CC coefficient value compared to the other group. As was mentioned in the methods section, we made two groups based on CC1 (CC1BC) and CC3 (CC3BC) to compare group 1 and group 2.

After classifying the data based on CC1 and CC3, the correlation analysis in (Table 1) was repeated, CC1BC results are shown in (Table 2). CC3BC provided similar results (see (Supplementary Table S2). A comparison of group 1 and group 2 in the terms of the association of fHRV with GA revealed that the r values were found to be higher in group 2 compared to group 1 (Table 2). Also, comparison between group 1 and group 2 revealed that maternal LF was found to be non-significantly related to GA in group 1 (Table 2).

**TABLE 2:** Comparison between group 1 and group 2 in terms of HRV and CC association with GA, *n* = 158.

Feature	Group 1 (low CC1)			Group 2 (high CC1)				
	Correlation between CC coefficients and GA							
	Median (min – max)	(Mean ± SD)	*r*	Median (min – max)	(Mean ± SD)	*r*		
CC1	0.43 (0.11–1.3)	0.47 ± 0.22	0.44†	0.67 (0.19–2.8)	0.72 ± 0.36	0.41†		
CC2	−0.042 (−0.86–0.81)	−0.064 ± 0.24	−0.21†	−0.035 (−1.8–1.2)	−0.10 ± 0.43	−0.16*		
CC3	0.40 (0.21–0.69)	0.41 ± 0.09	0.07	0.45 (0.22–0.71)	0.46 ± 0.09	0.17*		
CC4	−0.045 (−0.45–0.34)	−0.044 ± 0.15	−0.18†	−0.012 (- 0.53–0.49)	−0.021 ± 0.18	−0.13		
**Feature**	**Correlation between HRV and GA**							
	**Maternal Features**		**Fetal features**		**Maternal Features**		**Fetal features**	
	**(mean ± SD) median (min—max)**	***r***	**(mean ± SD) median (min—max)**	***r***	**(mean ± SD) median (min—max)**	***r***	**(mean ± SD) median (min—max)**	***r***
RRI (ms)	762 ± 116	−0.04	412 ± 26	0.38†	763 ± 115	−0.02	413 ± 24	0.40†
	761 (758–1,125)		408 (351–512)		763 (751–1,107)		410 (353–510)	
SDNN (ms)	33 ± 13	0.24†	15 ± 6.7	0.36†	36 ± 17	0.29†	16 ± 7.3	0.55†
	30 (10–87)		14 (4.5–35)		31 (13–120)		15 (4.0–45)	
SDHR (bpm)	3.5 ± 1.4	0.35†	5.5 ± 2.5	0.29†	3.7 ± 1.6	0.33†	5.7 ± 2.5	0.50†
	3.2 (1.3–9.2)		5.0 (1.2–14)		3.5 (1.5–11)		5.3 (1.6–14)	
VLF (Ln)	6.1 ± 0.81	0.36†	4.3 ± 1.2	0.30†	6.2 ± 0.80	0.34†	4.5 ± 1.1	0.52†
	6.1 (3.9–7.9)		4.4 (0.86–6.6)		6.2 (4.1–8.6)		4.6 (2.3–7.2)	
LF (Ln)	5.1 ± 0.80	0.09	4.3 ± 0.80	0.37†	5.1 ± 0.81	0.19*	4.3 ± 0.87	0.50†
	5.2 (2.6–7.2)		4.4 (1.9–5.9)		5.1 (3.3–7.7)		4.3 (1.4–6.5)	
HF (Ln)	4.8 ± 1.3	0.05	2.3 ± 0.77	0.49†	4.8 ± 1.3	0.10	2.2 ± 0.83	0.51†
	4.8 (0–8.0)		2.3 (0.67–4.2)		4.7 (1.1–7.8)		2.3 (−0.46–3.9)	

*p < 0.05, †*p* < 0.005. Abbreviations: HRV, heart rate (HR) variability; GA, gestational age; CC, cross-correlation; RRI, RR interval; SD, standard deviation; SDNN, SD of normal RRI; SDHR, SD of HR; bpm, beats per minute; VLF, ery low frequency power; LF, low frequency power; HF, high frequency power; *r*, spearman correlation coefficient. (The table was made based on CC1BC data set). Group1 has lower CC1 values compared to group 2, hence the “low CC1” and “high CC2” terms are added next to group 1 and group 2, respectively.

We conducted comparison of means tests to compare group 1 with group 2 twice, one comparison was based on CC1BC and the other was based on CC3BC, the results of the comparison are shown in (Table 3) (CC1BC in (Table 3) shows the same CC and HRV values as (Table 2). The results of the comparison between group 1 and group 2 showed that there were no significant differences in CC2 and CC4 (Table 3). Maternal VLF and SDNN were found to be significantly higher in group 2 compared to group 1 in both CC1BC and CC3BC (Table 3). SDNN is known to be significantly correlated with VLF (Shaffer and Ginsberg, 2017), hence, the significance observed in SDNN could be largely attributed to VLF. Maternal SDHR was found to be significantly higher in group 2 in the CC3BC.

**TABLE 3:** Comparison in medians between Group 1 and Group 2 (*n* = 158).

	*CC1BC*			*CC3BC*		
Feature	Group 1 median (min—max)	Group 2 median (min—max)	*P*—value	Group 1 median (min—max)	Group 2 median (min—max)	*P*—value
CC1	0.43 (0.11–1.3)	0.67 (0.19–2.8)	*p* < 0.01	0.47 (0.11–1.3)	0.58 (0.15–2.8)	*p* < 0.01
CC2	−0.042 (- 0.86–0.81)	−0.035 (-1.8–1.2)	0.87	- 0.032 (- 0.10–0.81)	−0.086 (−1.8–1.2)	0.11
CC3	0.40 (0.21–0.69)	0.45 (0.22–0.71)	*p* < 0.01	0.38 (0.21–0.65)	0.48 (0.27–0.71)	p < 0.01
CC4	−0.045 (−0.45–0.34)	−0.012 (−0.53–0.49)	1	−0.016 (−0.39–0.41)	−0.030 (−0.52–0.49)	0.43
**Maternal features**						
RRI (ms)	761 (758–1,125)	763 (751–1,107)	0.42	751 (537–1,125)	757 (530–1,107)	1
SDNN (ms)	30 (10–87)	31 (13–120)	0.026	30 (10–76)	33 (13–120)	0.039
SDHR (bpm)	3.2 (1.3–9.2)	3.5 (1.5–11)	0.17	3.2 (1.3–11)	3.6 (1.3–9.4)	0.031
HF (Ln)	4.8 (0–8.0)	4.7 (1.1–7.8)	0.13	4.7 (0–7.8)	4.7 (1.1–8.0)	0.81
LF (Ln)	5.2 (2.6–7.2)	5.1 (3.3–7.7)	0.17	5.0 (2.6–7.5)	5.1 (3.3–7.8)	0.69
VLF (Ln)	6.1 (3.9–7.9)	6.2 (4.1–8.6)	0.011	6.0 (3.9–8.0)	6.3 (4.1–8.6)	0.003
**Fetal features**						
RRI (ms)	408 (351–512)	410 (353–510)	0.87	411 (358–512)	407 (351–510)	0.002
SDNN (ms)	14 (4.5–35)	15 (4.0–45)	0.20	14 (4.0–45)	15 (4.5–36)	0.034
SDHR (bpm)	5.0 (1.2–14)	5.3 (1.6–14)	0.94	5.0 (1.2–14)	5.3 (1.7–14)	0.002
HF (Ln)	2.3 (0.67–4.2)	2.3 (-0.46–3.9)	0.81	2.4 (-0.46–4.1)	2.2 (0.46–4.2)	0.81
LF (Ln)	4.4 (1.9–5.9)	4.3 (1.4–6.5)	0.87	4.3 (1.4–6.3)	4.4 (1.6–6.5)	0.52
VLF (Ln)	4.4 (0.86–6.6)	4.6 (2.3–7.2)	0.63	4.3 (0.86–7.2)	4.6 (1.8–6.8)	0.11

Abbreviations: CC, cross-correlation; CC1BC, CC1 based classification; CC3BC, CC3 based classification; SD, standard deviation; RRI, RR interval; SDNN, SD of normal RRI; SDHR, SD of heart rate; VLF: very low frequency power; LF, low frequency power; HF, high frequency power.

With respect to fHRV, generally, there were less significant differences between group 1 and group 2. In CC3BC, fRRI was found to be significantly lower in group 2, whereas fetal SDNN and SDHR were found to be significantly higher in group 2.

## Discussion

We demonstrated the presence of similarities bmfRRITs. After performing a linear correlation analysis between the four CC coefficients and GA, the same similarities were found to be associated with fetal development (Table 1) and (Figure 4A). We showed that the similarities can be positive (Figures 3A–C), or negative (Figures 3D–F), and they may occur with a time lag. We speculate that the similarities bmfRRITs arise due to physiological processes regulated by the placenta such as oxygen and nutrition transfer. The presence of similarity bmfRRITs suggests that coordination between the mother and her child should exist for proper perfusion and exchange of blood supply through the placenta**.** The placenta is known to grow with GA, hence, regulations occurring within and affecting maternal and fetal HRs are expected to grow as well due to an increase in blood volume and fetal demand (Díaz et al., 2014). In our study, we found that our measure of similarity bmfRRITs, the four CC coefficients, increased in value with GA (Table 1) and (Figure 4A), also, we found that negative similarity trends dominate at advanced GA.

Due to the limited knowledge in the field, it is difficult to fully interpret the physiological differences between negative and positive similarities and this needs further research, but we believe that they could be related to fetal behavioral states and the typical fetal development cycle. fHRV is known to change with fetal behavioral states and the same states were found to change throughout gestation (Nijhuis et al., 1982; Pillai and James, 1990). Before 32 weeks of gestation, fetal activity has been classified into two states only which are activity and quiescency or resting (Pillai and James, 1990). In contrast, after 32 weeks of gestation, fetal activity was classified into four states which are: quiet sleep 1F, active sleep 2F, quiet awake 3F, and active awake 4F. In addition to fetal activity, negative and positive epochs could be related to the typical development cycle of fetal ANS. At early GA (<30 weeks), we expect the fetus to be more dependent on the mother for fHRV entrainment and ANS development. With fetal growth, the fetal dependency on the mother is expected to decrease and this may explain the increase in negative similarity epochs at advanced GA.

Fetal RRI and HRV were found to increase with GA (Table 1), and (Figure 4B), which indicates fetal development, our results are consistent with previous studies (David et al., 1985; Ohta et al., 1999; Laar et al., 2014; DiPietro et al., 2015; Amorim-Costa et al., 2017). Our results regarding fetal LF and HF are consistent with (David et al., 1985; Ohta et al., 1999) but different from what was reported by H. Gonçalves, et al. (2018). H. Gonçalves et al. (2018) showed that fetal LF and HF did not change with GA and the difference between their results and ours could be due to differences in the devices used for fHRV assessment. Here, we calculated HRV out of ECG, hence our HRV assessments were based on beat-by-beat calculations. On the other hand, H. Gonçalves et al. (2018) calculated HRV based on fHR measurements that were collected at a fixed rate of 4 Hz; fHRs were rounded to a quarter of a beat.

Among the mHRV features, only the maternal VLF, SDHR, and SDNN were found to increase significantly with GA (Table 1)and (Figure 4C). An increase in VLF with GA in pregnant women was also reported in previous studies (Walther et al., 2005; May et al., 2016; Mizuno et al., 2017). The increase in VLF with GA highlights its association with the regulation that causes the similarity bmfRRITs and this is further confirmed by the results in Table 3 in which it is shown that maternal VLF values are significantly higher in group 2 in both CC1BC and CC3CB. The physiological explanation of the VLF in pregnant women received little attention in previous literature. Also, in non-pregnant adults, VLF is considered less defined compared to HF and LF (Shaffer et al., 2014). The power within the VLF is believed to be associated with hormonal-related effects since they changed due to angiotensin-converting enzyme (ACE) inhibition (Akselrod et al., 1981; Taylor et al., 1998) and thermoregulation (Fleisher et al., 1996; Nkurikiyeyezu et al., 2017). Similarly, we expect that the maternal VLF during pregnancy could be associated with hormones that are critical for pregnancy-related regulations. As was mentioned before, the regulations are likely to be connected to the placenta.

The placenta lacks autonomic or neuronal innervations, hence, perfusion of blood through the umbilical cord is believed to be regulated by placental hormones (Díaz et al., 2014). Blood perfusion through the placenta and umbilical cord depends on vascular resistance and pressure (Myatt, 1992), and we expect that the regulation that causes the similarity plays a role in controlling them. The fact that maternal and fetal HR patterns share similarities given their separate ANS systems suggests the presence of mediating hormones that control both ANS, but exact identification of such hormones is elusive and more research is needed. We believe the estrogen to be related to the similarity and maternal VLF because estrogen is secreted by the placenta and increases with pregnancy weeks and it peaks in the third trimester (Robinsona and Kleina, 2013). The increase in estrogen with GA is consistent with our results regarding the increase of the CC coefficients and maternal VLF with GA [(Table 1), (Figure 4A), and (Figure 4C)]. Moreover, estrogen was found to play a critical role in the downregulation of ACE and upregulation of ACE 2 (Gallagher et al., 1999; Ojeda et al., 2007; Brosnihan et al., 2008), ACE 2 promotes vasodilation whereas ACE promotes vasoconstriction (Dhaundiyal et al., 2021). Previously, it has been reported that inhibition of ACE increased VLF (Akselrod et al., 1981; Taylor et al., 1998), hence, the increase in VLF in our study could be related to the increase in estrogen, and upregulation and downregulation of ACE 2, and ACE, respectively, but more investigation is needed to confirm this.

The similarities bmfRRITs imply that abnormal changes inflicted in the maternal cardiovascular system may eventually be reflected in fHRV, therefore, studying them is potentially critical for the assessments of fetal development, and pregnancy and birth outcomes. Understanding the patterns associated with the similarities may help uncover the causes behind some of the cardiovascular diseases that are believed to be related to the maternal uterine environment (Arima and Fukuoka, 2020). Also, the presence of similarities bmfRRITs suggests the need for developing clinical biomarkers based on both maternal and fetal HRs. The importance of developing biomarkers that depend on simultaneous records of maternal and fetal HR was also highlighted previously by H. Gonçalves et al. (2016) who performed a simultaneous analysis of maternal and fetal HR to identify fetal acidemia during labor.

According to the results in (Table 2), it is implied that the similarities may impact evaluations of fetal development based on short-term fetal RRI and HRV as the *r* values between both groups were different. Also, in (Table 3), there were differences in fetal RRI, SDRR, and SDHR values between both groups in CC3BC. Discrepancies between CC1 and CC3 in terms of differentiating fHRV parameters and RRI were found (Table 2), and this implies that different parameters are being measured by them. However, such differences do not negate the fact that similarities bmfRRITs impact fetal RRI and HRV. It is worth mentioning that we could base our classification on CC1 only because it had the highest correlation with GA with *r* = 0.40 [(Table 1); (Figure 4A)], however, we performed data classification based on CC3 as well since normalized CC coefficients are widely used in research compared to non-normalized CC coefficients.

In our study, we avoided classifying our data based on CC2 and CC4 due to the complexity involved in dividing the data and interpreting the results. CC2 and CC4 measure positive and negative similarity trends. In (Table 1); (Figure 4A), positive and negative trends were found to cluster before and after GA = 30 weeks, respectively. Hence, classifying data based on positive and negative trends is an indirect way of classifying data based on GA. The latter may provide a false interpretation of the connection of HRV with the similarity bmfRRITs. Also, a comparison between positive and negative groups may lead to a comparison between two subjects rather than two (5-min) epochs of the same subject. In CC1BC and CC3BC, we are comparing two 5-min segments that belong to the same subject, therefore, we are negating the effect of GA and other factors (e.g., maternal weight, age).

We used a 2-s window for CC analysis because we aimed at using the lowest optimal window size to capture detailed beat-by-beat variations induced simultaneously by fetal and maternal HRV. The maximum average value of mRRI was around 1,125 ms, hence we used a 2-s window to accommodate for the mRRI. Our conclusions regarding the increase in similarity bmfRRITs with GA (Table 1); (Figure 4A), were based on 5-min segments, hence, analysis of different time segments may provide different results. Also, different results could be obtained if different methods were used for similarity assessments. We collected our data in a supine position which is known to reduce HR (Watanabe et al., 2007; Li et al., 2019), hence it is unknown how similarities along with their assessments may change with body postures.

The retrospective design of the study constitutes the major limitation. Our sample consisted of subjects of different ages and pregnancy complications, which might have affected the correlations to some extent. Also, fetal behavioral states (Nijhuis et al., 1982; Gonçalves et al., 2007) and fetal gender (Bernardes et al., 2008; Gonçalves et al., 2017) are known to affect fHRV, therefore, they might have also affected the correlations as well. Hence, further studies are needed to study the effect of pregnancy complications, age, fetal gender, or fetal behavior on the similarities bmfRRITs and HRV. It is worth mentioning that we did not have further information regarding fetal gender because the data were anonymized for ethical purposes.

In conclusion, we discussed the presence of similarity bmfRRITs. The same similarity, which was quantified by using CC analysis, was found to be associated with fetal development. HRV analysis and data classification based on CC coefficients showed that maternal VLF is potentially associated with the similarity as well indicating that maternal hormones could be a major regulator of the similarities bmfRRITs.

## Data Availability

The datasets presented in this article are not readily available because ethical restrictions apply on sharing ECG data without proper request. Requests to access the datasets should be directed to Yoshiyuki Kasahara: kasa@med.tohoku.ac.jp.
