# Supplementary material for: Similarities between maternal and fetal RR interval tachograms and their association with fetal development
Source: Front Physiol. 2022 Nov 21;13:964755. doi: 10.3389/fphys.2022.964755 (PMC9721082; doi:10.3389/fphys.2022.964755)
Supplement: Supplementary file 1 [file DataSheet1.docx]

**Similarities between maternal and fetal RR interval tachograms and their association with fetal development**

Namareq Widatalla, Ahsan Khandoker, Mohanad Al Khodari, Kunihiro Koide, Chihiro Yoshida, Yoshiyuki Kasahara, Yoshitaka Kimura, Masatoshi Saito

**Supplementary Material**

Supplementary Table 1: Detailed information about the participants (n=172)

| **Maternal Condition** | **Number of cases** | **GA (weeks)**  median (min-max) | **Age (years)**  median (min-max) |
| --- | --- | --- | --- |
| Normal | 44 | 32 (20 – 39) | 34 (22 – 44) |
| Central nervous system (CNS) disease | 11 | 34 (23 – 38) | 32 (22 – 40) |
| Essential hypertension | 3 | 23 (21 – 27) | 29 (29 – 42) |
| Blood disease | 5 | 28 (23 – 38) | 41 (27 – 41) |
| Thyroid disease | 1 | 28 | 38 |
| Mental Illness | 13 | 31 (22 – 39) | 35 (27 – 40) |
| Respiratory disease | 9 | 31 (23 – 40) | 29 (27 – 38) |
| Gestational Diabetes | 5 | 33 (19 – 38) | 31 (23 – 34) |
| Uterine/appendix disease (UAD) | 19 | 28 (20 – 38) | 36 (29 – 43) |
| Autoimmune disease | 10 | 31 (25 – 39) | 33 (29 – 36) |
| Heart disease | 3 | 25 (23 – 26) | 26 (25 – 33) |
| Placenta previa | 6 | 34 (20 -36) | 37 (31 – 40) |
| Bone and muscle system disease | 2 | 28 & 32 | 33 & 33 |
| Kidney disease | 1 | 28 | 32 |
| Pre-eclampsia | 1 | 37 | 27 |
| Diabetes | 1 | 37 | 32 |
| Psoriasis vulgaris | 1 | 33 | 32 |
| Osler's disease | 1 | 36 | 32 |
| Cervical weakness | 1 | 39 | 30 |
| Blood disease & gestational diabetes | 1 | 33 | 41 |
| Cervical weakness & gestational diabetes | 1 | 22 | 41 |
| Kidney and respiratory diseases | 1 | 37 | 32 |
| UAD & autoimmune disease | 6 | 31 (26 – 38) | 33 (28 – 34) |
| UAD & Thyroid disease | 2 | 32 & 39 | 26 & 37 |
| UAD & digestive system disease | 1 | 20 | 41 |
| Thyroid and blood diseases | 1 | 23 | 37 |
| Thyroid and autoimmune disease | 1 | 24 | 30 |
| Autoimmune disease and placenta previa | 1 | 37 | 39 |
| Respiratory disease and mental illness | 1 | 20 | 38 |
| Mental illness and placenta previa | 1 | 37 | 32 |
| UAD & Mental illness | 3 | 34 (20 – 38) | 34 (25 – 34) |
| UAD & respiratory disease | 2 | 20 & 39 | 38 & 45 |
| UAD & Bone and muscle system disease | 1 | 23 | 44 |
| UAD & Urinary system disease | 1 | 38 | 39 |
| CNS & Bone and muscle system disease | 1 | 37 | 42 |
| Gestational diabetes & placenta previa | 1 | 33 | 30 |
| UAD & gestational diabetes | 1 | 26 | 34 |
| Respiratory disease & placenta previa | 2 | 35 & 37 | 35 & 39 |
| UAD & essential hypertension & thyroid disease | 1 | 38 | 41 |
| UAD & placenta previa | 1 | 33 | 32 |
| UAD & CNS | 1 | 23 | 35 |
| Placenta previa & cervical weakness | 1 | 25 | 35 |
| Heart disease and thyroid disease | 1 | 20 | 43 |
| Heart disease & respiratory disease and CNS | 1 | 2 | 27 |


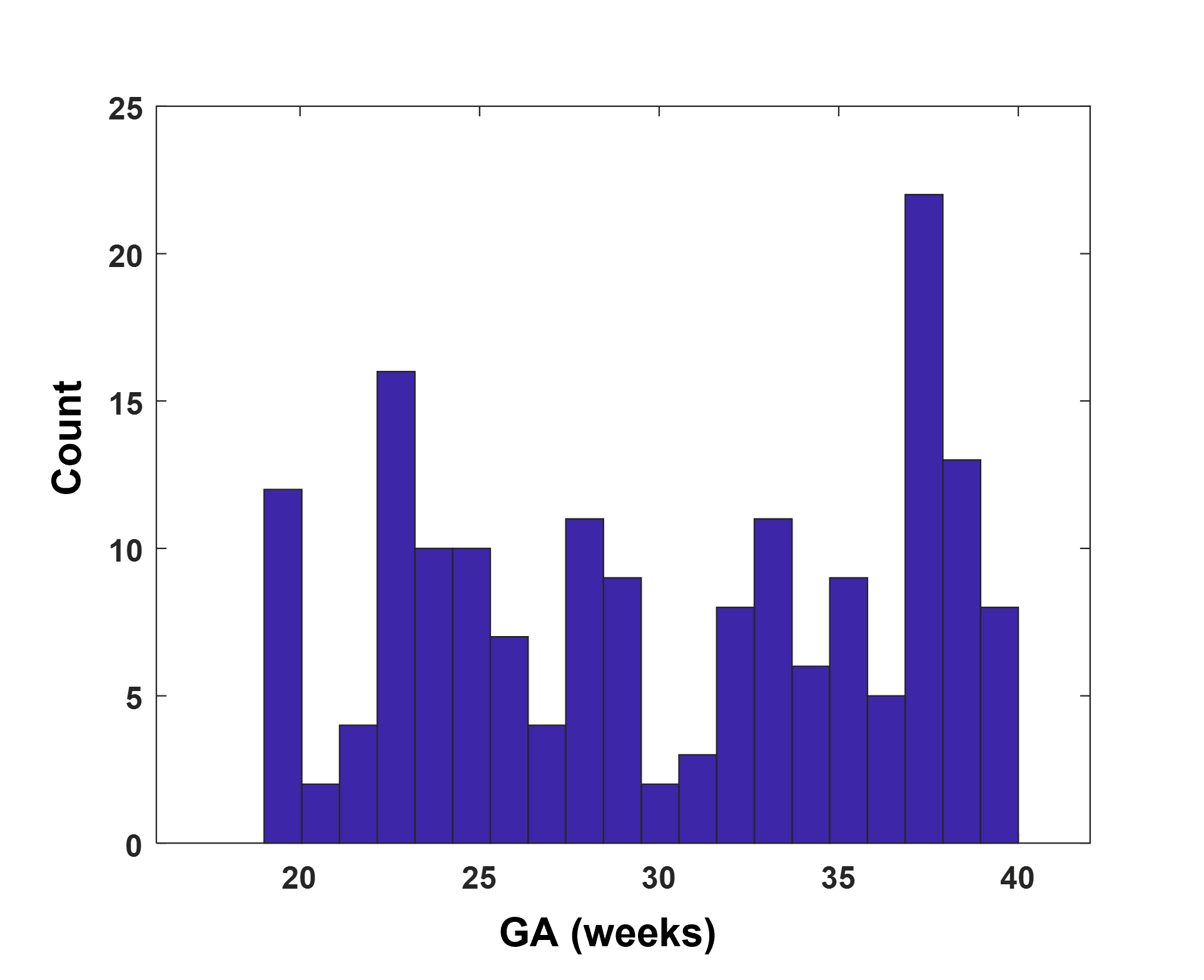


**Supplementary Figure 1:** Gestational age (GA) distribution of the data in supplementary table 1.


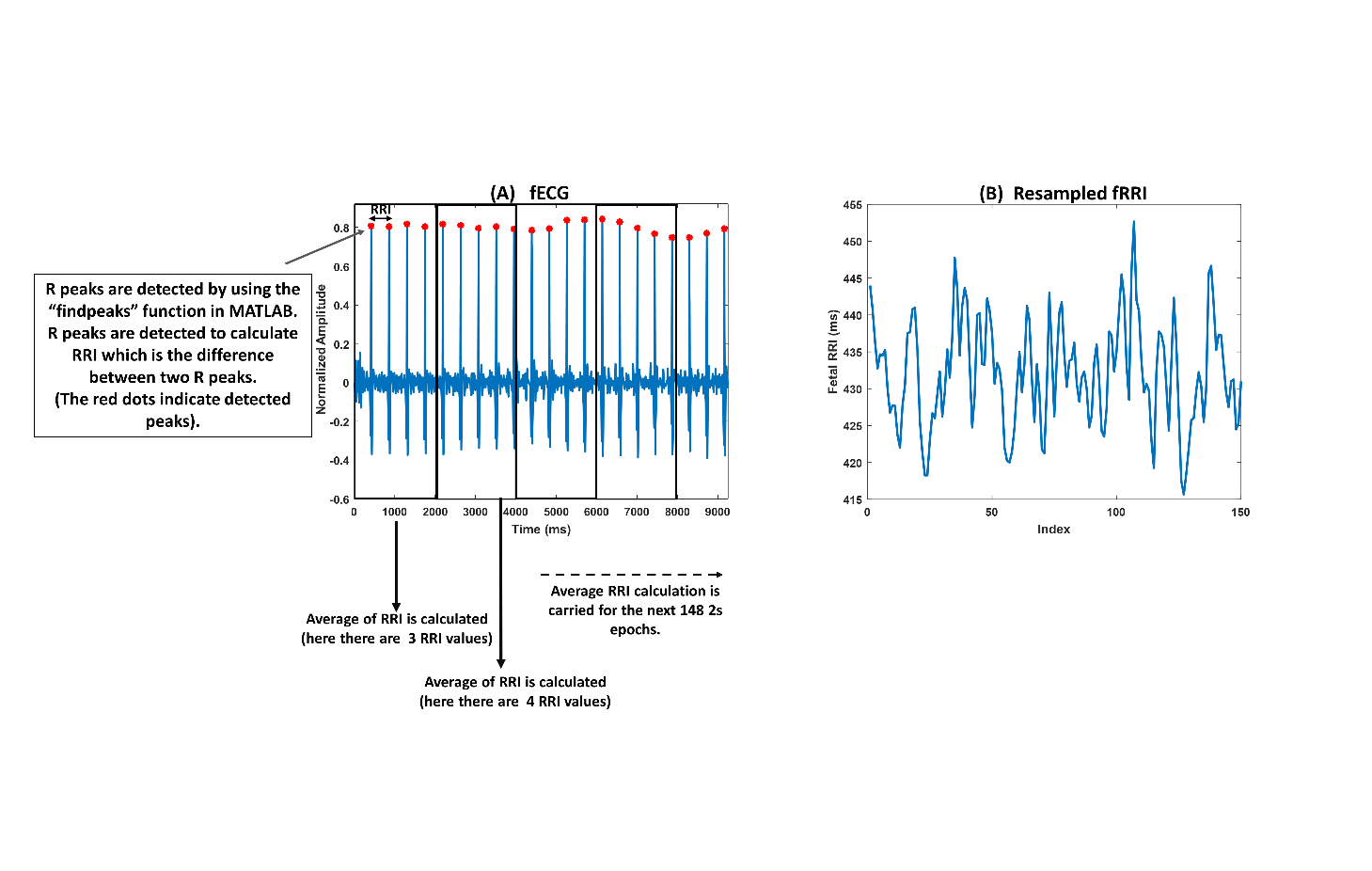


**Supplementary Figure 2: Example of RR interval (RRI) resampling at 0.5Hz (2 seconds).** (A) The figure shows the original fetal electrocardiogram (fECG) trace with detected R peaks (red dots). The average RRI was calculated per 2 second epoch. (B) the figure shows the resampled RRI.


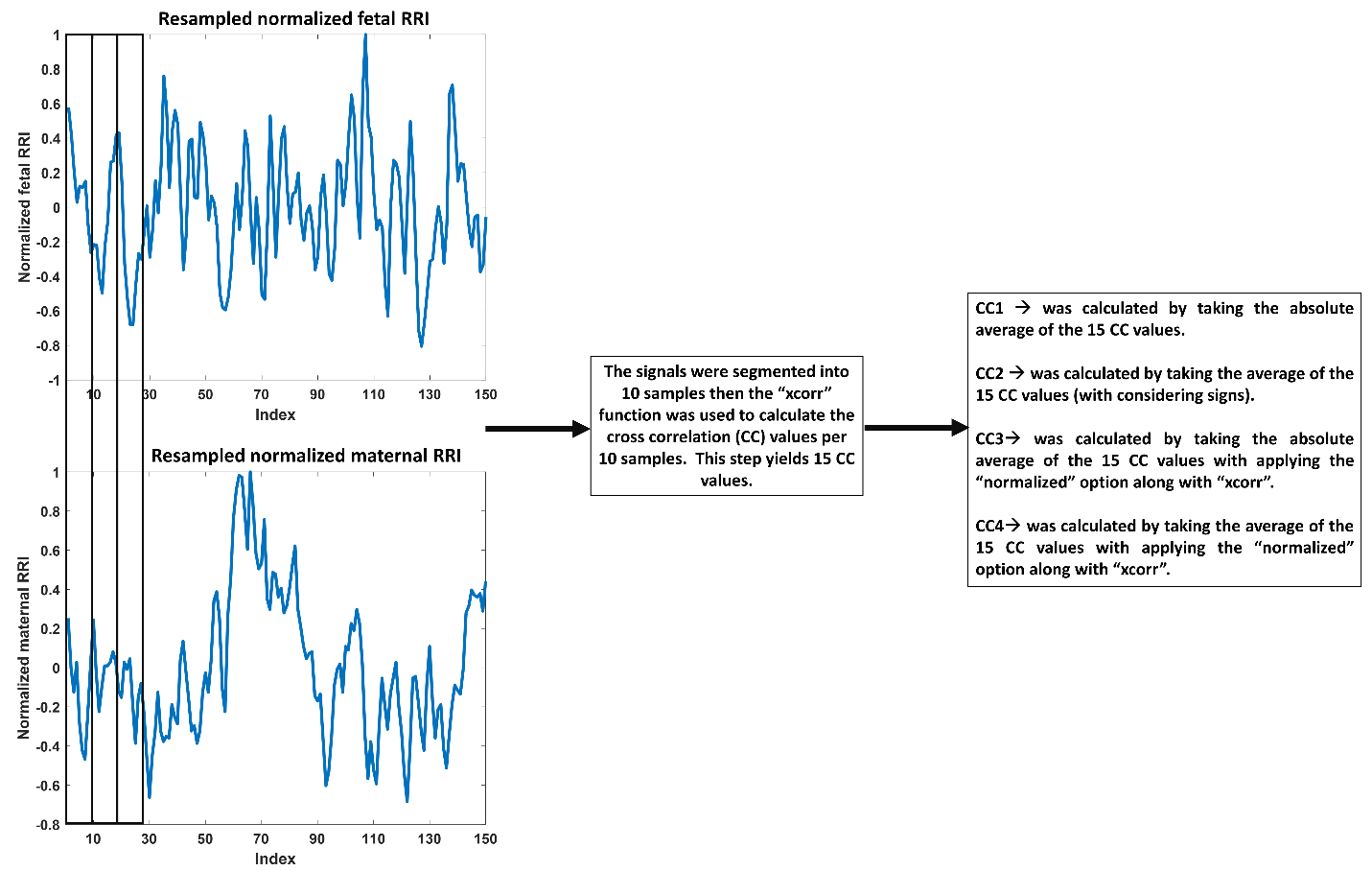


**Supplementary Figure 3: Cross correlation (CC) calculation.** The figure provides an illustrative summary of the steps that were followed to calculate CC1, CC2, CC3 and CC4 from resampled normalized maternal and fetal RR interval (RRI). (The figure of fetal RRI is the same as the one in supplementary figure 2B).

**Supplementary Table 2: Comparison between group 1 and group 2 in terms of HRV and CC association with GA, n=158**

|  | **Group 1 (Low CC3)** | | | | | | **Group 2 (High CC3)** | | | | | |
| --- | --- | --- | --- | --- | --- | --- | --- | --- | --- | --- | --- | --- |
| **Feature** | **Correlation between CC coefficients and GA** | | | | | | | | | | | |
|  | median (min – max) | | | (mean ± SD) | *r* | | | median (min – max) | | (mean ± SD) | *r* | |
| **CC1** | 0.47 (0.11 – 1.3) | | | 0.52 ± 0.24 | 0.40^†^ | | | 0.58 (0.15 – 2.8) | | 0.67 ± 0.37 | 0.40^†^ | |
| **CC2** | - 0.032 (- 0.10 – 0.81) | | | - 0.064 ± 0.24 | - 0.21^†^ | | | -0.086 (-1.8 – 1.2) | | - 0.12 ± 0.40 | - 0.16^*^ | |
| **CC3** | 0.38 (0.21 – 0.65) | | | 0.38 ± 0.08 | 0.13 | | | 0.48 (0.27 – 0.71) | | 0.48 ± 0.08 | 0.16 | |
| **CC4** | - 0.016 (- 0.39 – 0.41) | | | - 0.017 ± 0.14 | - 0.23^†^ | | | -0.030 (- 0.52 – 0.49) | | -0.05 ± 0.18 | - 0.11 | |
| **Feature** | **Correlation between HRV and GA** | | | | | | | | | | | |
|  | **Maternal Features** | | **Fetal features** | | | | | **Maternal Features** | | **Fetal features** | | |
|  | (mean ± SD)  median (min – max) | *r* | (mean ± SD)  median (min – max) | | | *r* | | (mean ± SD)  median (min – max) | *r* | (mean ± SD)  median (min – max) | | *r* |
| **RRI (ms)** | 763 ± 117  751 (537 – 1125) | - 0.06 | 414 ± 25  411 (358 – 512) | | | 0.42^†^ | | 762 ± 113  757 (530 – 1107) | - 0.01 | 411 ± 25  407 (351 – 510) | | 0.37^†^ |
| **SDNN (ms)** | 32 ± 13  30 (10 – 76) | 0.20^*^ | 15 ± 6.8  14 (4.0 – 45) | | | 0.37^†^ | | 37 ± 17  33 (13 – 120) | 0.33^†^ | 16 ± 7.2  15 (4.5 – 36) | | 0.57^†^ |
| **SDHR (bpm)** | 3.4 ± 1.4  3.2 (1.3 – 11) | 0.31^†^ | 5.3 ± 2.4  5.0 (1.2 – 14) | | | 0.32^†^ | | 3.8 ± 1.5  3.6 (1.3 – 9.4) | 0.39^†^ | 5.9 ± 2.6  5.3 (1.7 – 14) | | 0.52^†^ |
| **VLF (Ln)** | 6.0 ± 0.78  6.0 (3.9 – 8.0) | 0.34^†^ | 4.3 ± 1.2  4.3 (0.86 – 7.2) | | | 0.29^†^ | | 6.3 ± 0.81  6.3 (4.1 – 8.6) | 0.37^†^ | 4.6 ± 1.1  4.6 (1.8 – 6.8) | | 0.53^†^ |
| **LF (Ln)** | 5.1 ± 0.84  5.0 (2.6 – 7.5) | 0.08 | 4.3 ± 0.87  4.3 (1.4 – 6.3) | | | 0.35^†^ | | 5.1 ± 0.77  5.1 (3.3 – 7.8) | 0.19**^*^** | 4.3 ± 0.81  4.4 (1.6 – 6.5) | | 0.52^†^ |
| **HF (Ln)** | 4.8 ± 1.3  4.7 (0 – 7.8) | 0.04 | 2.3 ± 0.79  2.4 (-0.46 – 4.1) | | | 0.49^†^ | | 4.8 ± 1.2  4.7 (1.1 – 8.0) | 0.11 | 2.2 ± 0.79  2.2 (0.46 – 4.2) | | 0.53^†^ |

*** P < 0.05,** † **P <0.005, HRV: heart rate (HR) variability, GA: gestational age, CC: cross-correlation, RRI: RR interval, SD: standard deviation, SDNN: SD of normal RRI, SDHR: SD of HR, bpm: beats per minute, VLF: very low frequency power, LF: low frequency power, HF: high frequency power, *r*: spearman correlation coefficient. (The table was made based on CC3BC data set)**
